# To the Graduating Class

**Published:** 1913-05

**Authors:** 


### BUFFALO MEDICAL JOURNAL

**A Monthly Review of Medicine and Surgery**

EDITOR AND PUBLISHER DR. A. L. BENEDICT, 228 Summer St., corner of Elmwood Ave., Buffalo. (Address for all communications. Please make personal and telephone calls before 1 P. M.)

THE BUFFALO MEDICAL JOURNAL is the third, in age, in the western continent. It is independent but supports professional organization. As a scientific medium it is unrestricted in territory. As a news medium it is limited mainly to the western four districts of the Medical Society of the State of New York. The co-operation of physicians is asked in securing personal, obituary and other news items. Secretaries of Medical Societies in this region are requested to send brief reports of meetings. Full transactions will be published at cost of composition.

Subscription $2.00 a year in advance; $3.00 for two years in advance. Changes and errors in address or failure or duplication of delivery, should be reported immediately. Back numbers will be supplied, if possible but requests for copies should be made promptly.

YEARLY VOLUME 68. MAY, 1913 No. 10

*There are two kinds of ambition: to get the highest return for the least number of hours of work and the lowest expenditure of energy; and to give the greatest efficiency and to produce the best output. The choice between these marks the difference between the trade and the professional spirit.*

### To the Graduating Class

Years ago, when Associate Editor of the old, yellow-covered *Medical and Surgical Reporter* of Philadelphia, it was our custom to welcome the graduating class, in annual editorials, and to give advice, drawn largely from our own short-comings. In those days a seasonable article of this sort was possible, as the great majority of new recruits went into active practice at once, without preliminary interne service and with no delay on account of state boards. Conditions have changed for the better. Ninety per cent. of medical graduates now serve varying periods in hospitals and there is no definite season at which practice is begun. The graduates are also more mature, both in average years and in mental training, than formerly, and less in need of advice.

It is our intention, therefore, not so much to give advice, in the ordinary sense, as to mention certain conditions which may not have occurred to the medical man during his undergraduate course.

Education, general and technical, is very properly regarded as a criterion of the right to practice medicine and certain other professions. It is not everything, however. Honesty and integrity and devotion are also important—even in a purely business way. If there were an absolutely perfect medical school, which admitted only ideally educated men and gave them perfect familiarity with medical science and art and impressed on them the fact that the preparation was perfect, the result would be disastrous. Some of the greatest men in medicine are half educated, a few even so imperfectly educated that they cannot speak or write correctly—and we do not allude to foreigners—they are graduates of schools which gave one imperfect course in didactic medicine, with some clinics and a very little laboratory training, and turned them out to practice when they had gone over this course twice in sessions of less than six months. They had no hospital experience. But, they have succeeded, both from the economic and the scientific standpoint, because they realized their own short comings and have been taking post graduate courses of twelve months, every year, in their practice, in medical meetings, in medical journals, and in their own little laboratories. Some of these men—and others—have not only schooled themselves to make up for deficiencies in their early training for a life work but in their education as well. There is a club in many cities which admits no one but a graduate of a college in the limited sense. But, in every such city, you will find an equal number of men, of every profession and business represented, who are not eligible to admission in that club, but who are equally able, of equal standing and equally well informed.

What has impressed us more than anything else regarding professional success, in either scientific or economic sense, is its independence of the perquisites and the externals supposed to foster it. You are fortunate in that youth and lack of experience are less considered by the public as drawbacks than formerly. Men of extremely youthful appearance, with incipient mustache or no facial hirsutes at all, have been highly successful. So have men of insignificant physique, sissy manners and speech, dudish clothes, effeminate habits and all sorts of physical handicaps. On the other hand, it is incorrect to imagine that what ought to be a handicap is really an advantage. Most undergraduate students, in any kind of a school, are brought up in the belief that the man of low marks will ultimately roll in wealth while his scholarly associate will pass a life of genteel poverty. So far as early school days are concerned, the studies having only a remote bearing on life work, or even for a literary college where the idler is rather apt to be so because he has family backing and potential opportunities and where the man of high percentages is so because of a special adaptability to studies which will not be of direct service to him except as a teacher or professional scientist and because he has so little chance in life that he cannot afford to have a good time, there is something to be said as to the actuality of this notion. But, in the case of a medical school, in which scholarship is rated as nearly as is possible according to preparation for life work, we are very certain that ultimate success, even in an economic sense, will correspond fairly accurately to class standing. This opinion is based on actual investigation of the records of personal acquaintances. Of course, we do not mean to say that other factors than scholarship and especially of scholarship that can be figured in percentages, have no influence nor that the curves of class marks and of dollars of income will run parallel, but, taking groups of men of high and low standing in the medical school, the average degree of success in each group will correspond pretty closely to the group standing in college.

Contrary to the teachings of the fourth reader, we have been impressed with the fact that the average man keeps close to the intellectual and financial standing with which he starts. There are exceptions in both directions but the rule holds good. The fourth reader bases its contention largely on President Lincoln. Superficially considered, here is a typic case of a poor, handicapped youth, of the humblest parentage, reaching the height of success according to either low or high standards of judging success. But it is a mere matter of history that Lincoln belonged to one of the oldest and most aristocratic families in the country and that he simply regained and surpassed the position in life normal to his family, and which had been lost for a generation or two on account of the exigencies of pioneer life. Fortunately, there is not enough in heredity and early environment so that reasonable ambition backed by industry has not a good chance of success nor so that one may not lose an advantage with which he starts life.

There has been a great deal said as to the injustice of the failure in a practical sense of men of great ability and of the success of men who are merely bluffers. On the other hand, it has been held that practical success demonstrates whether the ability is real and whether the bluff is not merely the anticipation of a hand which the man deserves to hold and which he actually draws after the preliminary deal. We have been puzzling over these opposing explanations for some years and have not reached a conclusion that either is quite correct. Since assuming active work in connection with a medical journal, we have had a hint from a broader experience with different kinds of business men. So far as we can offer an explanation, it is this: In every occupation, including medicine, success means getting from about five up to a hundred or more times one's average share. Hence, in every recognized and established business or profession in which one is a candidate for success there is an over-supply of men for positions. In medicine there are easily two physicians for every practice of a magnitude to represent even relative, routine success. This is not a theory, but a fact, demonstrable by statistics in two or three independent ways, each corroborating the other. The conditions are probably worse for the law, but, without attempting to give figures, it may be said that, in all professions and businesses, which are at all desirable, the strain and effort are not so much to do one's work as to get it. Hence the great majority, even of conscientious, faithful, industrious and really able men, are relatively failures. Occasionally, in our own profession, as well as in other professions and businesses, one meets a man whose work comes to him easily and without conscious effort on his part, or even without especially strict and prompt attention to duty or any high degree of satisfaction to those actually coming to him as patients, clients or customers. In business such a man is the one who does not have to advertise—for there really are such—and in medicine he is the one who has unprecedented and undeserved "luck." But the real explanation of this power of attraction remains hidden; one can say what it is not but not what the peculiar power is.

It should also be clearly recognized that medicine, like journalism, most forms of art, teaching, the ministry, etc., has only a few opportunities for such signal success as may be found in law and in many forms of business. One may count on his fingers all the medical men in the country who have won success at all comparable with dozens in every line of business. Thus when, as frequently happens, a physician seizes an opportunity accidentally thrown in his way, to enter some other line of activity, it is usually said that he has failed in medicine. The truth of the matter is that no one fails in anything, so long as he hangs onto the rope.

Another fact of importance, especially when we consider the high suicide rate of the medical profession (over 7 per cent. of all deaths), is the falsity of the old saw that "Opportunity knocks once at every man's door." The fact is that Opportunity calls every little while and that it would be most unwise to take up her first offer simply because of the belief that there will never be another such a bargain offered. And, on the other hand, it should not be refused if it is really a bargain, with the hope of beating her down to better terms later. Opportunity may knock at your door early with a chance to secure "success" by marrying a rich girl. If you seize the chance as a matter of business, you may find that the opportunity is about the same as giving up medicine for a butler's position, with the advantage that you will eat at table with the family and the disadvantage that you will have no time off. In other cases, while the arrangement will be pleasant enough socially, the incentive to hard work will be absent and the opportunities for ultimate professional success will fritter away from mere lack of necessity. You do not imagine that the possession of money or any other good fortune implies unworthiness of either a woman or a man.

Opportunity may come in the form of a government position or that of assistant to some successful practitioner. In such a case you will have to solve the difficult problem of estimating accurately your opportunities for independent success. If you ask advice your adviser may underestimate your real chances or he may exaggerate them. Don't simply aim high. Aim to hit the mark, estimate your own projectile power as well as you can. One bit of advice may be given with assurance: Don't take any kind of a position in which you are to be "used" without definite assurance of recompense, of an adequate degree, subject, of course, to "making good." And, don't expect a genuine return from anything in which you engage simply for notoriety and prestige and not with the idea of performing a genuine and necessary work.

